# Trait variation in response to varying winter temperatures, diversity patterns and signatures of selection along the latitudinal distribution of the widespread grassland plant *Arrhenatherum elatius*

**DOI:** 10.1002/ece3.2936

**Published:** 2017-04-09

**Authors:** Stefan G. Michalski, Andrey V. Malyshev, Juergen Kreyling

**Affiliations:** ^1^ Department of Community Ecology (BZF)Helmholtz Centre for Environmental Research UFZHalleGermany; ^2^ Institute for Botany and Landscape EcologyErnst Moritz Arndt University GreifswaldGreifswaldGermany

**Keywords:** adaptive divergence, genetic diversity, genotyping‐by‐sequencing, latitudinal gradient, *Q*_ST_‐*F*_ST_ comparison, winter climate

## Abstract

Across Europe, genetic diversity can be expected to decline toward the North because of stochastic and selective effects which may imply diminished phenotypic variation and less potential for future genetic adaptations to environmental change. Understanding such latitudinal patterns can aid provenance selection for breeding or assisted migration approaches. In an experiment simulating different winter temperatures, we assessed quantitative trait variation, genetic diversity, and differentiation for natural populations of the grass *Arrhenatherum elatius* originating from a large latitudinal gradient. In general, populations from the North grew smaller and had a lower flowering probability. Toward the North, the absolute plastic response to the different winter conditions as well as heritability for biomass production significantly declined. Genetic differentiation in plant height and probability of flowering were very strong and significantly higher than under neutral expectations derived from SNP data, suggesting adaptive differentiation. Differentiation in biomass production did not exceed but mirrored patterns for neutral genetic differentiation, suggesting that migration‐related processes caused the observed clinal trait variation. Our results demonstrate that genetic diversity and trait differentiation patterns for *A*. *elatius* along a latitudinal gradient are likely shaped by both local selection and genetic drift.

## 1 Introduction

In the face of global climatic changes that alter local environmental conditions at unprecedented rates, studies investigating the ability of species and populations to cope with such changes are urgently needed (Cahill et al., 2013). Directional selection may very quickly lead to adaptive genetic changes (Franks, Sim, & Weis, 2007; Jump et al., 2008), but often the rate of current environmental change outpaces rates of adaptation (Wilczek, Cooper, Korves, & Schmitt, 2014). As a consequence, local extinction and/or migration toward more suitable habitats are expected to dramatically shift current distribution ranges (Parmesan, 2006; Thomas et al., 2004). Understanding trait variation and the underlying genetic basis along environmental gradients can help to project the ecological consequences of climate change and resulting population dynamics and, hence, species distributions (Chevin, Lande, & Mace, 2010). The potential for in situ adaptation of populations depends on two factors: First, the ability to react via microevolutionary processes given by the degree of genetic diversity in phenotypic traits, that is, the heritability and the speed at which selection favors genetic changes. Heritable variability in quantitative traits, however, can change in response to the environment in which it is expressed (Hoffmann & Merilä, 1999; Sgrò & Hoffmann, 2004). As a consequence, adaptive responses may vary among different environments even if the strength and direction of selection for a trait expression are similar. Second, the ability of individuals to express different phenotypes in different environments (Ghalambor, McKay, Carroll, & Reznick, 2007). In fact, this phenotypic plasticity may bridge the time period necessary for genetic adaptations (Chevin et al., 2010). Furthermore, if plasticity itself is a genetically controlled trait, it can be expected to respond to changing selective pressures if more plastic genotypes have a fitness advantage over less plastic genotypes (Van Kleunen & Fischer, 2005).

Genetic diversity may vary across a species range because of stochastic processes known as genetic drift as well as adaptive processes in response to differential selection regimes. In general, population genetic diversity can be largely influenced by both historical and contemporary changes to population size and gene flow (Eckert, Samis, & Lougheed, 2008; Frankham, 1996; Vucetich & Waite, 2003). For example, in Europe, rapid northward expansion from southern, topologically varied refugia and (re)colonization of habitats after the Last Glacial Maximum have led to a reduction in neutral genetic diversity from lower to higher latitudes in many species (Hewitt, 1996, 1999). Via its impacts on effective population size selection may also act as a factor which reduces genetic diversity (Corbett‐Detig, Hartl, & Sackton, 2015) and harsher environments in the North or at the range edge possibly exert stronger selection regimes than elsewhere in the distribution range. Generally, spatially heterogeneous site conditions may have selected for locally adapted genotypes throughout a species distribution range (Hufford & Mazer, 2003; Joshi et al., 2001). In addition, also phenotypic plasticity may vary within a species’ distribution due to differently acting selection pressures. In particular, higher environmental heterogeneity has been hypothesized to favor broader ranges of environmental tolerances and acclimation responses (Ghalambor, Huey, Martin, Tewksbury, & Wang, 2006; Janzen, 1967). This may result in clines of plasticity if this heterogeneity changes with latitude, climatic conditions, or toward the range periphery of a species (Lazaro‐Nogal et al., 2015; Molina‐Montenegro & Naya, 2012). Comparing genetic differentiation patterns at neutral marker loci with those at traits under selection using equivalent measures is a promising approach to understand to what degree the differential response of populations is caused by adaptive versus stochastic processes (Leinonen, McCairns, O'Hara, & Merila, 2013; Merila & Crnokrak, 2001). This knowledge is increasingly in demand not only for fundamental research but also for applied strategies in restoration and conservation of biodiversity (Carroll et al., 2014; Sgrò, Lowe, & Hoffmann, 2011).

Climate change in Europe is expected to result in increasing mean temperatures, but future scenarios may differ regionally. For example, temperature variability is expected to increase nearly everywhere, but more strongly in Central Europe (Schär et al., 2004), and annual winter temperatures are expected to increase more rapidly at higher latitudes (IPCC 2013, P1350, Figure AI.36). Low temperature is arguably the single most limiting factor influencing natural plant distributions (Parker, 1963) and can also significantly affect yield of forage crops in northern temperate regions (Ouellet, 1976). Depending on the current mean winter temperatures, sites with temperatures close to but below freezing will experience the largest decreases in soil freezing days (Henry, 2008) with a higher chance of complete absence of frost in lower latitudes (Henry, 2013; Kreyling & Henry, 2011). Nonetheless, random frost events are still predicted to occur with similar intensity and duration in the future (Kodra, Steinhaeuser, & Ganguly, 2011) and can be especially damaging to plants when they occur following a period of warm weather (Bokhorst, Bjerke, Tømmervik, Callaghan, & Phoenix, 2009). Moreover, snow cover is projected to strongly decrease across Europe with climate warming (Kreyling & Henry, 2011), resulting in more variable or even colder conditions close to the soil surface due to decreased insulation (Kreyling, 2010). Therefore, across the latitudinal distribution of a species, in particular if it is widespread, different future winter scenarios are possible but difficult to predict locally.

**Figure 1:**
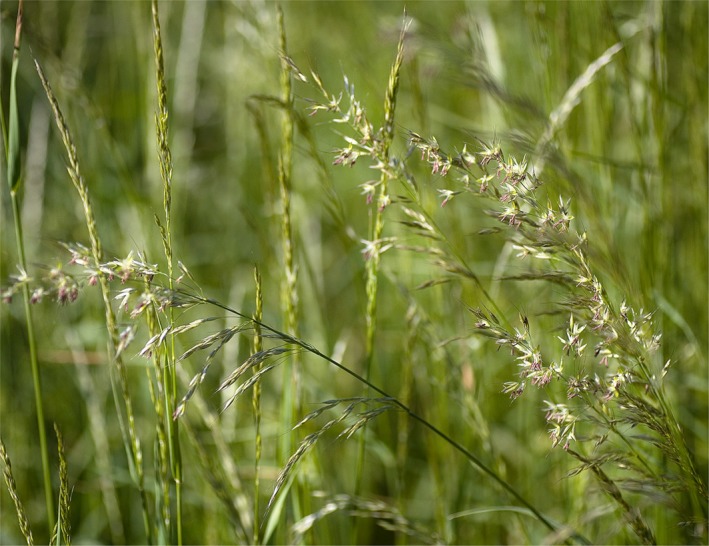
Flowering inflorescences of *Arrhenatherum elatius* in a meadow dominated by the species

Here we assess genetic variability and phenotypic plasticity in quantitative traits of the grass *Arrhenatherum elatius* (L.) P. Beauv. ex J. Presl & K. Presl (Poaceae, Figure 1). The species is a common and widespread plant in Central European semi‐natural grassland areas, which, as the second half of the 20th century, are affected by more rapidly occurring, regionally differing land‐use changes (Hejcman, Hejcmanova, Pavlu, & Benes, 2013) threatening grassland‐associated high biodiversity levels. We ask whether the response of *A*. *elatius* to different mid‐winter scenarios differs with latitude of sample origin and whether the differences can be attributed to adaptive or neutral processes. Previous studies on *A. elatius* found high phenotypic and genetic variability for quantitative traits (Jenkin, 1931; Mahmoud, Grime, & Furness, 1975; Petit & Thompson, 1998; Sulinowski, 1965) as well as adaptive differentiation at different spatial scales (Kreyling et al., 2012; Petit & Thompson, 1998; Voeller et al., 2012). Further, investigations at the molecular level revealed comparatively high levels of genetic diversity within populations, negatively correlated with the number of growing degree days (Michalski et al., 2010). Differentiation as response to climatic conditions is likely to contribute to the overall genetic differentiation patterns (Durka et al., 2017; Michalski et al., 2010). Here, in a common garden experimental setup, we compared accessions of *A. elatius* originating from along a latitudinal gradient in Europe, also covering a large spatial and climatic gradient. Mid‐winter climate manipulation scenarios consisted of (1) mild temperatures without frost, (2) an extended frost period, and (3) relatively warm temperatures followed by a short, sudden frost. The three mid‐winter climate scenarios were designed to simulate different possible mid‐winter conditions occurring across the locations of seed origins, that is, frozen soil toward the North, unfrozen soil toward the South, and random high‐temperature variability occurring across the latitudinal distribution. We assessed quantitative trait variation under the different experimental conditions and compared diversity and differentiation patterns between quantitative and putatively neutral molecular traits. More specifically, we asked (1) whether quantitative trait expression and genetic diversity, and the plastic response to the different mid‐winter treatments show latitudinal clines, and (2) whether the observed quantitative trait differentiation shows signatures of selection.

## 2 Materials and Methods

### 2.1 Study species

*Arrhenatherum elatius* is a very common, relatively drought resistant forage grass. It is an autotetraploid, wind‐pollinated perennial, and has been described as primarily self‐incompatible with a low degree of self‐fertility (Cuguen, Acheroy, Loutfi, Petit, & Vernet, 1989). The species is native in and widely distributed throughout Europe but introduced into North America, New Zealand, and Australia. In Europe, the limits of its northern distribution range coincide with the −6.7°C January isotherm (Pfitzenmeyer, 1962). The species may not be native in large parts of its current distribution range in Europe but introduced following an increase in cultivation of grasslands at the end of the Middle‐ages or even later (Buch, Hetzel, Loos, & Keil, 2007; Conert, 1998). However, fossil and other evidence support an earlier, indigenous distribution in Central and Northern Europe (Hejcman et al., 2013; Kauter, 2002; Roehrs, Klooss, & Kirleis, 2013). Today, the species is frequently included in seed mixtures for pastures and used in restoration measures.

### 2.2 Quantitative experiments

#### 2.2.1 Plant material and plant cultivation

**Figure 2:**
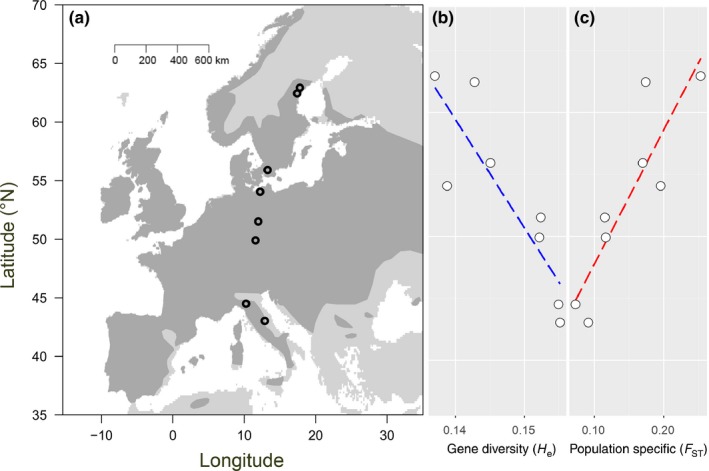
(a) Locations of sampled populations of *Arrhenatherum elatius* across Europe. The area shaded in dark gray represents the actual distribution of the species. (b) Latitudinal decline of molecular genetic diversity (sample size corrected *H*_e_ averaged across 5,026 SNP loci, Pearson correlation *r* = −.87, *p *=* *.005). (c) Increase in population‐specific molecular differentiation (*F*_ST_) with latitude (*r* = −.89, *p *=* *.003). Regression lines (dotted) have been plotted for visualization of the trends

In 2012, open‐pollinated seed families were collected from eight locations in Europe representing a latitudinal gradient from 43.03°N to 62.94°N (see Figure 2, Table S1) and kept dry in paper bags. At regional level, locations were chosen arbitrarily from available semi‐natural grasslands not recently restored or created. Seed families were sampled arbitrarily at the respective locations, hence representing the local population. From each location, 15 offspring from five seed families were raised for a total of 600 plants. This sample size was chosen as a compromise between technical feasibility, that is, space limitation in the growth chambers (see below) and representation ability. Also, we were more interested in describing latitudinal patterns than in estimating absolute population characteristics, which possibly would require larger sample sizes. Individuals were cultivated at the Leibniz Institute of Plant Genetics and Crop Plant Research in Poel, Germany. Plants were germinated for 3 weeks on filter paper in a climate chamber set at close to 100% humidity, with a 12‐h photoperiod, and mean day and nighttime temperatures of 22 and 15°C, respectively. Seedlings were then transplanted into seed compost soil (Classic Profisubstrate, Einheitserde, Germany) in plastic pots (5 cm diameter × 7 cm deep). An NPK‐(Mg) fertilizer (Hakaphos Blau, COMPO Expert, Germany) was applied once with each plant receiving a total amount of approximately 6 mg. Plants were transferred to a greenhouse where day and night temperatures averaged 19.6°C, SE: ±0.9°C, and 9.6 ± 0.9°C, respectively, and a 10‐hours photoperiod was provided with 400 W lamps. Plants grew for another 5 weeks and were trimmed twice, with the final trimming to a height of 2 cm prior to the acclimation treatments. Plants were trimmed to standardize plant height at the start of the experiment as well as to prevent shading of neighboring plants by leaves of bigger plants. Acclimation was conducted in Bayreuth, Germany, in climate chambers with a light intensity of 180 μmol m^−2^ s^−1^.

#### 2.2.2 Plant acclimation and winter temperature treatments

Plant cold acclimation was initiated 11 weeks after sowing by lowering the temperature to 4°C and reducing the photoperiod to 9 hr. All plants experienced the same cold acclimation conditions for 25 days (temperature averaged 4.7 ± 0.1°C) before undergoing one of three simulated mid‐winter scenarios chosen to approximate common temperature regimes in Southern, Northern and Central Europe, respectively (Figure S1): (1) a frost‐free (mild) winter scenario with plants kept at an average hourly temperature of 2.6 ± 0.3°C for 8 days, (2) a constant low‐frost scenario (frost) with plants frozen at an average temperature of −3.8 ± 0.1°C for 8 days, and (3) a temperature fluctuation scenario (warmfrost) with plants kept at an average of 8.0 ± 0.1°C for 6.5 days before being frozen down to −4°C for 1 day. Plants within each treatment were kept in trays of 50 plants (4 per treatment), and all trays were arranged randomly in one chamber during the cold acclimation phase (hereafter referred to as the block effect nested within the treatment effect), alternating positions within the chamber every 2 days. For the three winter treatments, a separate climate chamber was used for each treatment, where the tray positions were again altered as mentioned above. At the conclusion of the mid‐winter treatments, plants were repotted into larger pots (8 cm × 8 cm × 20 cm) using the same soil and transferred to a greenhouse where temperatures averaged 17.0 ± 0.2°C for the next 2 months. At all stages of the experiment, individuals were arranged completely randomized. Taken together, the experiment artificially comprised a shortened full annual cycle with spring germination, summer growth, fall acclimation, variable mid‐winter temperatures, onset of growth in spring, and summer flowering.

#### 2.2.3 Response traits

Growth performance following frost exposure has previously been used as a relative measure to evaluate the effectiveness of plant cold acclimation (Malyshev & Henry, 2012). Here, we recorded three vegetative growth traits (1) initial plant height measured 2 days after ceasing the different mid‐winter treatments, (2) final plant height 4 weeks after the treatments, (3) relative change in plant height, measured as the difference between final height and initial height, and (4) above‐ground biomass measured 5 weeks after the treatments. Biomass was cut at 2 cm above ground allowing regrowth. None of the plants had formed inflorescences yet. Our biomass harvest can be considered a natural disturbance, as the grass is frequently grazed and/or mown under conditions in the field. Plant material was dried at 60°C to a constant biomass and weighed. Height measurements were taken as the average height of three leaves per plant. Also, as a fifth response trait taken after regrowth, each plant was monitored for the presence or absence of inflorescences approximately 2 months after the biomass harvest (flowering probability).

### 2.3 Molecular genetic analysis

Leaf material from one offspring per seed family was sampled, and DNA was extracted from freeze‐dried material with the DNeasy 96 Plant extraction kit (QIAGEN, Hilden, Germany). Assessment of molecular diversity and differentiation patterns was carried out using SNP data obtained by a genotyping‐by‐sequencing approach (GBS). SNP genotyping was conducted at the Genomics Diversity Facility of Cornell University, Institute of Biotechnology, USA. In short, the GBS libraries were constructed using the PstI restriction enzyme and a protocol modified from Elshire et al. (2011) and sequenced on an Illumina HiSeq 2000/2500 (100 bp, single‐end reads). The whole library was sequenced twice to increase the coverage per locus. The GBS UNEAK analysis pipeline (Tassel Version: 3.0.174) was run with default options, except for a minimum minor allele frequency cutoff in the HapMap file set to 0.01. The pipeline was run using a script which allows retrieving exact read counts per sample and locus (Mimee et al., 2015). From the bi‐allelic SNP data retrieved, codominant genotypes were called using a maximum‐likelihood approach as follows: Assuming that the observed number *k* of reads for allele A (of *n* reads, with *n *≥* *2) follows a binomial distribution with parameters *n* and *p*, where *p* is the unknown frequency of allele A in the tetraploid genotype (i.e., *p* = 0, 0.25, 0.5, 0.75, and 1), we computed the value of *p* that maximizes the likelihood of *k*. Using likelihood ratio tests (at an α = .05 level) between the likelihood for the best *p* and likelihoods for all other possible *p* values, we obtained a measure of confidence for the best *p*. In case of nonsignificant comparisons, we called incomplete genotypes (e.g., A000, AB00, or ABB0). Eventually, for further analysis only polymorphic loci scored for a minimum of two samples per population were retained. Molecular genetic diversity across loci was calculated at population level as gene diversity corrected for sample size (*H*_e_, Nei, 1978) using SPAGeDi v1.5 (Hardy & Vekemans, 2002). To obtain a neutral differentiation estimate (*F*_ST_), we screened the data for loci putatively under selection using BayesScan 2.1 (Foll, Fischer, Heckel, & Excoffier, 2010; Foll & Gaggiotti, 2008) and default options. Loci with a q‐value lower than 0.05 were assumed to be non‐neutral and excluded from *F*_ST_ calculation. Global and pairwise *F*_ST_ estimates were obtained using the Weir–Cockerham approach (Weir & Cockerham, 1984) implemented in SPAGeDi v1.5. Population‐specific *F*_ST_ values (all loci) were obtained from BayesScan 2.1.

### 2.4 Data analysis

#### 2.4.1 Diversity patterns along latitude

We used generalized linear mixed models to explain the observed trait variability using latitude of sample origin, mid‐winter treatment, and their interaction as fixed factors. Sample origin, seed family, and their interaction with treatment as well as block were entered as random effects. Model selection was based on the REML implementation in the package “lme4” (Bates, Maechler, Bolker, & Walker, 2013) for R (R Core Team 2012). We optimized the random structure on the full fixed effect model first (minimum AIC) and subsequently assessed individual‐fixed effects by stepwise inclusion and likelihood ratio tests (Zuur, Ieno, Walker, Saveliev, & Smith, 2009, p.121ff). Estimates were obtained by fitting best models in a Bayesian framework implemented in the package “MCMCglmm” (Hadfield, 2010) as well as by REML. Information on prior and run specifications as well as convergence checking can be found in the Supporting Information. We tested for latitudinal effects on population estimates for molecular genetic diversity and differentiation, and heritability in quantitative traits with simple Pearson correlations.

Heritability (*h* ^2^) for each trait and population was estimated (a) across all experimental conditions and (b) for each condition separately. Therefore, models were fitted in “MCMCglmm” (a) with treatment as fixed, block and seed family, and the family x treatment interaction as random effects and for (b) with only block and seed family as random terms. Total phenotypic variance explained (*V*_T_) was partitioned into the additive genetic variance (*V*_A_) and the residual variance (*V*_R_) composed of the error variance and the variance explained by block and for a) additionally the variance for the family × treatment interaction. Heritability (*h* ^2^) was then defined as the ratio *h* ^2^ = *V*_A_/*V*_T_ = *V*_A_/(*V*_A_ + *V*_R_) (Petit et al., 2001). Assuming a half‐sib experimental design, *V*_A_ was calculated from the variance among seed families (*V*_F_) as *V*_A_ = 4*V_F_ (Gallais, 2003). Total phenotypic variation for the binary trait flowering probability included an additional term accounting for the variance introduced by the logit link function, that is, π^2^/3. We did not quantify heritability of plasticity as none of the best models (see below) included a significant seed family × treatment interaction. If the collected open‐pollinated seed families consist indeed of half‐sibs only, the quantity *h* ^2^ can be interpreted as narrow‐sense heritability. However, as the relatedness among sibs has not been assessed directly, our heritability estimates may include nonadditive and additionally maternal effects. Heritability as a measure of evolutionary potential has been criticized in general, and mean‐scaled additive genetic variances (evolvability) have been proposed as more suitable instead (Hansen, Pélabon, & Houle, 2011). Hence, we also calculated evolvability as *e* = 4*V_a_/m^2^, where m is the trait mean, and repeated all analyses with this measure.

#### 2.4.2 Differentiation patterns

Quantitative genetic differentiation in mean traits among populations (*Q*_ST_) was estimated across all experimental conditions, and for each condition, separately fitting models in “MCMCglmm” containing treatment as fixed or only the intercept, respectively, and block, population, and seed family as random effects. The total genetic variance was partitioned into among (*V*_P_) and within‐population (*V*_A_) components (represented by four times the among‐family variance because of the half‐sib design), and genetic differentiation was quantified as *Q*_ST_ = V_p_/(2*V_A_ + V_p_) (Lande, 1992; Spitze, 1993). This definition is derived for diploids and may be different for autotetraploids, for which a proper equivalent is still lacking (Bever & Felber, 1992). We are still confident in our results involving *Q*_ST_ calculations (see. Section 2.4.3) and do not draw absolute conclusions based on these alone. *Q*_ST_ values were estimated first at the overall level including all populations and second from all pairwise comparisons between populations. We also quantified *Q*_ST_s of plasticity running models including additional random terms estimating variances for the treatment × population and treatment × family interaction. *Q*_ST_ of plasticity was then estimated from these variance components as described above. Precision estimates for global *Q*_ST_ values were directly derived from the posterior distribution as suggested by O'Hara and Merilä (2005). To test for a significant isolation‐by‐distance or isolation‐by‐climate pattern, estimates of pairwise neutral genetic (*F*_ST_) and quantitative differentiation were tested against pairwise spatial and climatic distances among populations. We tested also for covariation between quantitative and neutral genetic differentiation. Significance of the correlations was evaluated by (partial) Mantel tests in R with 1,000 permutations. Climatic distances between sampled populations were obtained as Euclidean distances from scaled bioclimatic data (19 variables) for each sample location extracted from the Worldclim database [http://www.worldclim.org/] (see also Figure S2).

#### 2.4.3 Tests for adaptive differentiation and plasticity

Adaptive divergence and signatures of selection can be detected by comparing differentiation at quantitative traits with that expected under neutrality (Leinonen et al., 2013). To test whether quantitative genetic differentiation in mean traits and plasticity (*Q*_ST_) differed from neutral expectations, we followed the approach of Whitlock and Guillaume (2009) by reporting the difference between the observed *Q*_ST_ and a simulation expected under neutrality ($Q_{\mathrm{ST}}^{n}$). Hence, a deviation from zero directly indicates divergent or homogenizing selection in case of a significant positive or negative deviation, respectively. A distribution of $Q_{\mathrm{ST}}^{n}$ values was calculated by simulating a neutral among‐population variance 1,000 times as $V_{P}^{n}$ = 2*F_ST_ * V_A_/(1‐*F*_ST_) and then multiplying by a factor *r*/(npop‐1), with npop the number of populations considered and *r* being a random number drawn from a chi‐square distribution with npop‐1 degrees of freedom, to simulate the sampling distribution around this expectation. For each simulation, the *F*_ST_ value obtained from GBS data was used, and a V_A_ value was sampled from the posterior distribution of the models described above. $Q_{\mathrm{ST}}^{n}$ values were then computed using the observed within‐population variance. The test statistic was calculated as the difference between 1,000 *Q*_ST_ values drawn from the posterior distribution of the model and 1,000 simulated $Q_{\mathrm{ST}}^{n}$ values and considered to be significant if the 95% credible interval did not include zero.

To further evaluate the adaptive significance of the plastic response to the different mid‐winter scenarios, we used an approach described by Hahn, van Kleunen, and Mueller‐Schaerer (2012). First, as approximations for fitness, the overall trait means at seed family level were estimated across treatments for biomass and probability of flowering. Second, an overall relative plasticity index at seed family level was computed as the coefficient of variation among treatment‐specific family means. Linear models were used to explain fitness by the plasticity index. The trait mean was included as additional term to separate the fitness effect from the absolute value. Hence a significant positive or negative slope suggests adaptive or maladaptive plasticity, respectively.

## 3 Results

### 3.1 Latitudinal patterns

#### 3.1.1 Mean trait response

**Figure 3:**
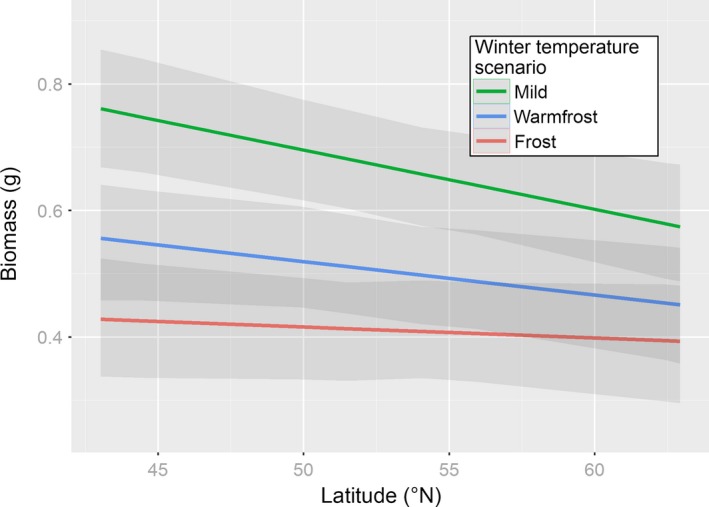
Biomass production of *Arrhenatherum elatius* (shaded gray: 95% credible interval) in relation to latitude responded differently to varying winter temperature conditions as predicted by a Bayesian mixed effect model

GLMM analyses revealed different response patterns for the traits investigated (Table 1). Except for the probability of flower production, mean trait expression differed significantly in response to the different mid‐winter treatments and hence, showed significant plastic behavior. With increasing latitude of sample origin, the mean probability of flowering decreased irrespective of the mid‐winter treatment (Figure S3). In contrast, the effect of latitude on biomass depended on the treatment (latitude × treatment interaction, Table 1, Figure 3). Based on the 95% credible intervals for slope estimates, plants from lower latitudes produced significantly more biomass than plants from higher latitudes after the mild than after the frost treatment. Estimates were nearly identical for both the MCMC and REML approaches (data not shown).

**Table 1:** Summary of Bayesian mixed‐effects model analyses evaluating the effect of simulated mid‐winter temperatures and latitude of sample origin and its interaction on plant biomass, height, relative change in height (growth rate), and flowering probabilty. For each best model, estimates for intercept and slopes with 95% credible intervals in parenthesis are shown for the fixed effects (treatment and latitude and latitude × treatment interaction, respectively), estimated standard deviations are given for the random effects. The difference in model fit to and, in parenthesis, the next best fixed effect model is indicated by ∆DIC

Trait	Fixed effect							Random effect						
	Latitude	Mid‐winter treatment			Latitude × treatment			Location ×					Family ×	
	Slope/intercept	Mild	Frost	Warmfrost	Mild	Frost	Warmfrost	Block	Location	treatment	Family	Residuals	treatment	∆DIC
Biomass	–	1.17 (0.90–1.43)	0.50 (0.22–0.76)	0.79 (0.51–1.04)	−0.009 (−0.014 to −0.005)	−0.002 (−0.006 to 0.003)	−0.005 (−0.010 to −0.001	0.07	–	–	0.07	0.19	–	3.5 (‐Lat × treatment)
Initial height	–	6.63 (6.40–6.88)	6.18 (5.92–6.42)	7.34 (7.09–7.57)	–	–	–	–	–	–	0.60	1.18	–	0.1 (+Lat)
Final height	–	19.90 (17.79–21.82	15.10 (13.03–17.06)	18.08 (16.10–20.06)	–	–	–	1.70	1.40	0.17	0.40	3.35	–	0.1 (+Lat)
Growth rate	–	2.05 (1.78–2.35)	1.53 (1.20–1.79)	1.53 (1.23–1.82)	–	–	–	0.28	–	–	0.17	0.57	–	0.6 (+Lat)
Flowering	−0.14 (−0.25 to −0.04)/6.08 (1.06 to 11.86))	–	–	–	–	–	–	–	1.24	–	–	1 (fixed)	–	3.4 (+treatment)

#### 3.1.2 Plasticity

Tests for the adaptive significance of relative plasticity for the different mid‐winter treatments revealed that plasticity in final height negatively affected fitness approximated by biomass production (*t* value = −2.265, *p *=* *.029) indicating maladaptive plasticity. Relationships for all other combinations of fitness approximations and trait plasticities were nonsignificant.

#### 3.1.3 Molecular and quantitative genetic diversity

**Figure 4:**
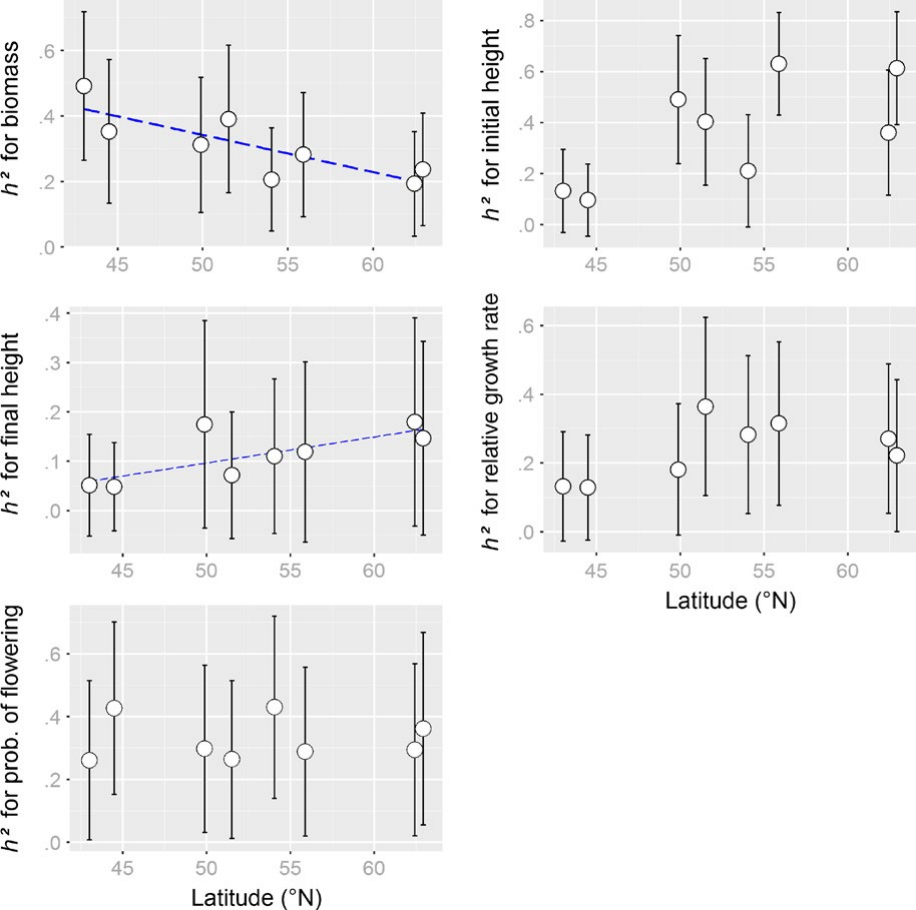
Latitudinal patterns for estimates of narrow‐sense heritability of vegetative growth variables and flowering in *Arrhenatherum elatius*. For visualization, a linear regression line (dashed, blue) is plotted for significant correlations. Error bars indicate the standard deviation of the estimates taken directly from the posterior distribution

For 37 of 40 samples, genotypic information could be obtained. For these samples, the GBS approach yielded 5,026 polymorphic SNP loci of which 51 were putatively under selection and hence, excluded from the neutral *F*_ST_ calculation. Population estimates for molecular genetic diversity and differentiation (all loci) showed a significant decline and increase with latitude, respectively (Figure 2). Across treatments, heritabilities for biomass significantly declined with increasing latitudes (*r* = −.83, *p = *.01, Figure 4) and showed (marginally) positive trends for initial and final plant height (*r* = .67, *p *=* *.07, and *r* = .75, *p *=* *.03, respectively). Heritabilities estimated for the different experimental winter conditions separately revealed an opposite trend for final height in the warmfrost scenario (*r* = −.72, *p *=* *.04) but no other significant correlations (*p ≥ *.1). Using evolvability instead of heritability, significantly negative correlations with latitude were found for biomass only (*r* = −.79, *p *=* *.02, and *r* = −.76, *p *=* *.03 for the across‐treatment and for the warmfrost estimates, respectively).

### 3.2 Differentiation patterns

Neutral genetic differentiation at the molecular level (*F*_ST_) was moderate as estimated from GBS data (*F*_ST_ = 0.090). Pairwise neutral molecular differentiation estimates did not follow an isolation‐by‐distance or an isolation‐by‐climate pattern (Mantel *p* > .33).

**Figure 5:**
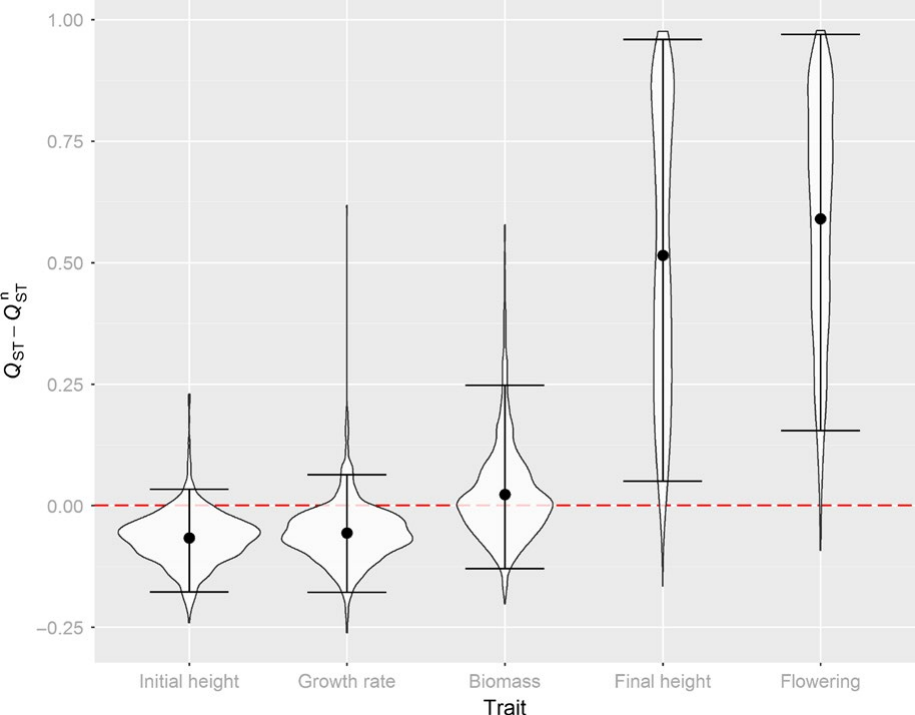
Violin plots of the difference between the posterior distribution of *Q*_ST_ and the simulated distribution of $Q_{\mathrm{ST}}^{n}$ for trait means indicating potential adaptive differentiation. The dashed, red line represents the zero difference between *Q*_ST_ and $Q_{\mathrm{ST}}^{n}$, that is, the expectation under a gene flow‐drift‐only scenario. The dots indicate the mean and the error bars the 95% credible interval of that difference

Across‐treatment quantitative genetic differentiation (*Q*_ST_, 95% credible interval) varied substantially among traits (biomass: *Q*_ST_ = 0.11, 0.01–0.29, initial height: *Q*_ST_ = 0.02, 0.00–0.09, final height: *Q*_ST_ = 0.60, 0.15–1.00, growth rate: *Q*_ST_ = 0.03, 0.00–0.11, and probability of flower production: *Q*_ST_ = 0.68, 0.25–1.00). Differentiation for final plant height and flowering probability significantly exceeded neutral expectations suggesting a contribution of diversifying selection (Figure 5). This pattern was not altered for initial height, growth rate, and biomass when the different experimental winter conditions were analyzed separately (Figure S4). However, for flowering probability, a higher *Q*_ST_ was expressed under the frost compared to the mild and warmfrost scenario, whereas the pattern was reversed for final height. Across‐treatment pairwise quantitative genetic differentiation between populations did not show an isolation‐by‐distance pattern (Mantel *p *<* *.27) but showed an isolation‐by‐climate pattern for initial and final plant height only (partial *r* = .63, Mantel *p *=* *.002 and partial *r* = .46, Mantel *p *=* *.02, respectively).

**Figure 6:**
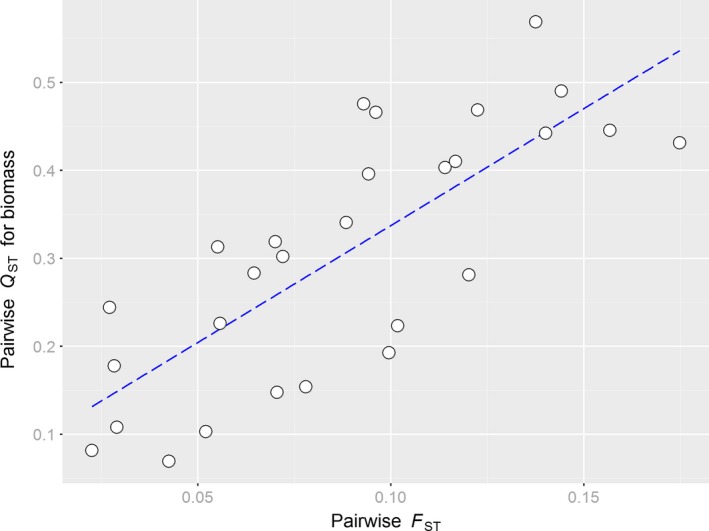
Significant positive correlation (*r* = .78, Mantel *p *=* *.001) between pairwise neutral genetic (GBS ‐*F*_ST_) and quantitative genetic differentiation (*Q*_ST_) for biomass among populations of *Arrhenatherum elatius* across a latitudinal gradient. For visualization of the trend, a regression line (dotted blue) has been plotted

Relating pairwise *Q*_ST_ values against pairwise neutral molecular differentiation values revealed a significant correlation for biomass only (*r* = .78, Mantel *P *=* *.001, Figure 6). Variance components estimated for assessing genetic differentiation in plasticity were close to zero for most traits, yielding *Q*_ST_ posterior distributions largely reflecting the prior information, that is, dumbbell‐shaped distributions with maxima toward zero and one (data not shown). We hence choose not to interpret results from this analysis.

## 4 Discussion

### 4.1 Adaptive trait variation

#### 4.1.1 Biomass

Genetically controlled latitudinal clines in biomass accumulation have been observed by common garden studies of numerous herbaceous plant species (e.g., Chapin & Chapin, 1981; Kollmann & Bañuelos, 2004; Li, Suzuki, & Hara, 1998; Woods, Hastings, Turley, Heard, & Agrawal, 2012) including many grasses (De Frenne et al., 2012; Leva, Aguiar, & Premoli, 2013; Novy, Flory, & Hartman, 2013). Among other environmental factors, latitude is strongly correlated to average temperature, solar radiation, and water availability which may act as strong selective agents resulting in smaller and less productive ecotypes in the North (Chapin & Chapin, 1981; Li et al., 1998). Our finding that heritability for biomass production was lower in higher latitudes may reflect the result of a stronger historic selection reducing within‐population additive genetic variance for that trait. Similarly, in an annual legume, less heritable trait variation in the North was found which was additionally correlated with decreased fitness (Etterson, 2004). It is generally expected that toward the range edge of a species, genetic diversity is reduced which is supported by our molecular data. This reduction, however, can be caused not only by a more severe selective pressure in less favorable environments, but also by increased effects of drift as an result of a more recent migration history and stronger bottlenecks which also should increase genetic differentiation at the periphery (Eckert et al., 2008; Sexton, McIntyre, Angert, & Rice, 2009). Also, for colonizing species, it has been shown that significant geographical clines in a quantitative trait can arise through serial founder effects alone (Colautti & Lau, 2015). Indeed, we showed that for biomass production, genetic differentiation among populations was very similar to neutral expectations (Figures 5 and 6), and pairwise population differentiation did not show a relationship to climatic distances as expected if trait divergence would have been the result of environment‐driven adaptive processes. Thus, the observed latitudinal clines for biomass production and heritability in *A*. *elatius* are more likely the result of neutral, migration‐related processes.

#### 4.1.2 Plant height and relative growth rate

An increase in relative growth rates toward the North might be expected to compensate for a shorter growing season (Robertson & Ward, 1970; Sawada et al., 1994). However, our results did not show a strict linear increase with latitude of origin neither for growth rate nor for initial or final plant height, suggesting that other factors may contribute to height‐related trait variability. At a smaller, regional scale, stem height in *A*. *elatius* was found to be adaptive in response to light intensity, that is, individuals from populations of more sun‐exposed habitats grew taller than individuals from darker habitats (Petit & Thompson, 1998). Also in our experiment, some pieces of evidence suggest adaptive trait variation and divergence for plant height. First, the latitudinal clines for heritable within‐population variation estimated across treatments for height traits suggest stronger selection on height in southern latitudes. Interestingly, this cline changed direction for final height in the warmfrost treatment rendering the interpretation of these clines a challenging task. Second, at least for final height, genetic differentiation among populations was stronger than expected under neutral population divergence. Further, genetic trait differentiation in height traits increased with climatic distances. In our experimental setup, initial plant height represents plant growth during winter, whereas final plant height reflects growth at the beginning of the growing season. It can be hypothesized that differences in the strength of overall differentiation between initial and final height are possibly shaped by the impact of selective influences of biotic interactions (cf. Welk, Welk, & Bruelheide, 2014), such as the competition for light and resources or plant‐herbivore relations, which are likely to be less important during winter.

#### 4.1.3 Flowering probability

Sexual reproduction in *A*. *elatius* has been described as severely handicapped in colder climates (Pfitzenmeyer, 1962). In our experiment, flowering probability strongly decreased with increasing latitude suggesting a genetic basis for this observation. Similar results were obtained by Malyshev, Henry, and Kreyling (2014), showing that northern ecotypes of *A*. *elatius* had always an equal or lower flowering probability as compared to southern ecotypes independent of photoperiod and temperature treatments during the cold acclimation phase. Indeed, a shorter growing season in the North may constrain flowering and investment to sexual reproduction (Olsson & Ågren, 2002; Quilot‐Turion et al., 2013). Although with only limited empirical support, it has also been hypothesized that fitness should be reduced toward the range edge (Abeli, Gentili, Mondoni, Orsenigo, & Rossi, 2014; Sexton et al., 2009). Hence, also indicated by the strong differentiation among populations, adaptive processes are very likely to have shaped the observed latitudinal flowering pattern. However, in our experiment, flowering was recorded in the first growing season after germination. It cannot be excluded that allocation in sexual reproduction increases for northern populations as plants grow older (cf. Olsson & Ågren, 2002).

#### 4.1.4 Trait plasticity

We found a significant plastic response to the different winter conditions in all measured traits except for flowering probability. In general, harsher or more variable winter conditions negatively affected regrowth in the following season. This response differed among populations for biomass production and final height indicating an overall genotypic effect on absolute plasticity. However, variation among genotypes within populations in the response to the different winter conditions, that is, a significant genotype × environment interaction (G × E) was not detectable in the traits assessed. It has been argued that under current and historical environments, selection keeps the reaction norm equally adaptive, and only in new or stressful environments a significant G × E effect might manifest (Ghalambor et al., 2007; Rutherford, 2000). Our winter treatment encompassed relatively moderate temperature conditions well within the natural environmental range of the species and all populations sampled. Thus, the treatment was probably not stressful enough to express genetic variation for plasticity which might facilitate adaptive evolution (Ghalambor et al., 2007). Additionally, autopolyploid plant species such as *A*. *elatius* are thought to exhibit a high degree of phenotypic homeostasis, at least compared to related diploid lineages because of an increased level of heterozygosity. This may enable polyploids to exploit a larger range of environmental conditions (Lowry & Lester, 2006) but may also buffer against fast microevolutionary changes. However, differences in the plastic response to environmental differences could not be detected between the diplopid and tetraploid lineages in *A*. *elatius* (Petit, Thompson, & Bretagnolle, 1996) or the similar grassland perennial *Dactylis glomerata* (Bretagnolle & Thompson, 2001).

### 4.2 Heritability and evolutionary potential

Our estimates for the heritable fraction of variation in biomass, height, and relative growth rate and flowering probability in *A*. *elatius* are well in line with other estimates for vegetative and reproductive growth parameters across a wide range of species (Geber & Griffen, 2003). For *A*. *elatius*, a number of studies, mostly conducted at regional scale in the center of the species distribution, have demonstrated significant heritable within‐population variation for morphological and phenological traits (Ducousso, Petit, Valero, & Vernet, 1990; Mahmoud et al., 1975; Petit & Thompson, 1997, 1998) or heavy‐metal tolerance (Gartside & McNeilly, 1974). Although significant heritability is a prerequisite for microevolutionary responses to environmental changes, the quantification of the evolutionary potential across large spatial and thus environmental scales based on estimates of heritability and genetic correlations from common garden experiments is likely afflicted with a high degree of uncertainty (Mitchell‐Olds & Rutledge, 1986). First, heritability and genetic correlations may change drastically from one environment to another if the underlying genetic basis is shifting (Sgrò & Hoffmann, 2004) which can partly be seen also in our study (see Supplementary material). Second, heritability estimates from common garden studies are found to be upwardly biased compared to estimates obtained in the wild (Geber & Griffen, 2003), where the relative importance of biotic interactions on trait expression is likely to be higher. Eventually, the evolutionary response of a certain trait depends on the actual additive genetic covariance with fitness (Price, 1970; Robertson, 1966) which can be very difficult to estimate (Morrissey, Kruuk, & Wilson, 2010). Additionally, predicting a species’ response to different winter conditions is even more complex as a number of interacting factors such as lengths of the photo‐ and growing period and plant pathogens may influence cold acclimatization and winter survival (Rapacz et al., 2014). Ultimately, in order to predict an evolutionary response to altered environments, quantitative genetic parameters should be assessed in natural populations, which, despite available approaches, requires still substantial efforts (Stinchcombe, 2014).

### 4.3 Practical implications

Semi‐natural grasslands hold an important part of biodiversity in terms of habitats and species and are a source for a wide range of ecosystem services (Dengler, Janišová, Török, & Wellstein, 2014). Common grassland species such as *Arrhenatherum elatius* are frequently used in commercial seed mixtures for both agriculture and ecological restoration measures. To avoid negative consequence of maladaptation at seeding sites, many authors are arguing for the use of local or regional seed sources in such approaches which is increasingly adopted by practitioners (Kiehl, Thormann, & Pfadenhauer, 2006; McKay, Christian, Harrison, & Rice, 2005; Vander Mijnsbrugge, Bischoff, & Smith, 2010). Hence, knowledge on genetic diversity and differentiation patterns and the relation to phenotypic variation and local adaptation is increasingly in demand in order to aid present‐day strategies for revegetation (Breed, Stead, Ottewell, Gardner, & Lowe, 2013; Kettenring, Mercer, Reinhardt Adams, & Hines, 2014).

At a more regional scale, that is, using a transplant experiment across Germany, Bucharova et al. (2017) found no evidence for adaptation in *A*. *elatius* contrasting to other grassland species investigated.

At the larger spatial scale, we demonstrate clinal trait variation which very often is adaptive (e.g., Kawakami et al., 2011; Walisch, Colling, Bodenseh, & Matthies, 2015). Although, clinal variation alone might be insufficient to prove regional adaptation as it can caused by migration‐related processes as evidenced by our results on biomass production. Still, our differentiation patterns for plant height and flowering probability still indicate adaptive responses, suggesting that for species such as *A*.* elatius* with a strong potential for gene flow, source zones still are advocated but could be delineated rather liberally.

In general, as a consequence of southern glacial refugial areas present‐day populations in the South may still harbor a larger pool of genetic diversity compared to more northern populations (Hampe & Petit, 2005). For *A*. *elatius*, the genetic impoverishment and increased drift effects at the molecular level toward the North are very likely the imprint of these historical processes. Hence, Southern Europe could be a preferred area to select lineages for breeding or as source for assisted migration approaches (Kreyling et al., 2011; Vitt, Havens, Kramer, Sollenberger, & Yates, 2010).

## Conflict of Interest

None declared.

## Data Archival Location

The raw data (quantitative and SNP data) underlying the main results of the presented study have been deposited at the dryad repository (doi:10.5061/dryad.1b5t7).
