# Supplementary material for: Trait variation in response to varying winter temperatures, diversity patterns and signatures of selection along the latitudinal distribution of the widespread grassland plant Arrhenatherum elatius
Source: Ecol Evol. 2017 Apr 9;7(9):3268–80. doi: 10.1002/ece3.2936 (PMC5415536; doi:10.1002/ece3.2936)
Supplement: Supplementary file 1 [file ECE3-7-3268-s001.docx]

**Supplementary material**

Trait variation in response to varying winter temperatures, diversity patterns and signatures of selection along the latitudinal distribution of the widespread grassland plant *Arrhenatherum elatius*

Stefan G. Michalski^1^, Andrey V. Malyshev^2^ and Juergen Kreyling^2^

*Information on priors, run specifications and diagnostics for MCMCglmm models*

All models in MCMCglmm (Hadfield 2010) were run with a normal prior for the fixed effects. An inverse Wishart prior for the random effects was parameterized with V = 1 and the degree of believe parameter mu = 0.002; a choice frequently used for variance components (Gelman 2006) leading to a weakly informative prior when the variance components are not close to zero. For models on flowering probability (binary data) the residual variance was fixed (Nakagawa and Schielzeth 2010), and for the random effects a ­Χ² prior distribution was implemented using the parameter extension possibility with V = 1, mu=1000 and the supplementary parameters alpha.mu and alpha.V set to 0 and 1, respectively. Convergence and mixing of chains was checked visually and by plotting running means for each estimated variance component and the number of iterations, burnin lengths and thinning was adjusted accordingly (see below). Autocorrelation was below 0.1 in all cases except for some variance components in models for assessing *Q*_ST_’s for plasticity (see methods).

**Run parameters for the different models**

Diversity patterns along latitude

*Trait variability ~ Latitude*

| *Trait mean* | *# of iterations* | *Burnin* | *Thinning* |
| --- | --- | --- | --- |
| Biomass | 240.000 | 40.000 | 100 |
| Initial height | 440.000 | 40.000 | 200 |
| Final height | 240.000 | 40.000 | 100 |
| Growth rate | 240.000 | 40.000 | 100 |
| Flowering probability | 2.400.000 | 400.000 | 1000 |

*Heritabilities*

For all traits and populations, across treatments and for each treatment separately, models were run with the same specifications for number of iterations, burnin and thinning, i.e. 1200000, 200000, and 500 respectively.

Differentiation patterns

*Global quantitative genetic differentiation in mean traits*

| *Trait mean* | *# of iterations* | *Burnin* | *Thinning* |
| --- | --- | --- | --- |
| Biomass | 250.000 | 50.000 | 100 |
| Initial height | 400.000 | 200.000 | 100 |
| Final height | 1.200.000 | 200.000 | 500 |
| Growth rate | 1.200.000 | 200.000 | 500 |
| Flowering probability | 2.400.000 | 400.000 | 1000 |

*Pairwise quantitative genetic differentiation in mean traits*

All models were run with the same specifications for number of iterations, burnin and thinning, i.e. 220000, 20000, and 100 respectively.

*Quantitative genetic differentiation in trait plasticities*

| *Trait plasticity* | *# of iterations* | *Burnin* | *Thinning* |
| --- | --- | --- | --- |
| Biomass | 450.000 | 50.000 | 200 |
| Initial height | 1.100.000 | 100.000 | 500 |
| Final height | 2.200.000 | 200.000 | 1000 |
| Growth rate | 1.100.000 | 100.000 | 500 |
| Flowering probability | 1.100.000 | 100.000 | 500 |

Family mean traits

Across treatment mean family traits were obtained from models including seed family as fixed, block and mid-winter treatment as random effect. Mean traits for each treatment separately were obtained analogous. Models for all traits were run with the same specifications for number of iterations, burnin and thinning, i.e. 220000, 20000, and 100 respectively.

**Table S1**. Sampled populations (from south to north) and molecular genetic diversity and differentiation estimates measured as sample size corrected gene diversity (*H*_e_) and population specific *F*_ST_ values based on GBS data.

| Population | Latitude (°N) | Longitude (°E) | *H*_e_ | *F_ST_* |
| --- | --- | --- | --- | --- |
| Colfiorito, Italy | 43.03 | 12.88 | 0.155 | 0.092 |
| Rusino, Italy | 44.50 | 10.26 | 0.155 | 0.074 |
| Destuben, Germany | 49.91 | 11.58 | 0.152 | 0.117 |
| Halle (Saale), Germany | 51.52 | 11.97 | 0.152 | 0.116 |
| Rostock, Germany | 54.06 | 12.23 | 0.139 | 0.196 |
| Trolleholm Castle, Sweden | 55.91 | 13.27 | 0.145 | 0.170 |
| Alnö, Sweden | 62.45 | 17.41 | 0.143 | 0.174 |
| Kramfors, Sweden | 62.94 | 17.79 | 0.137 | 0.253 |

**Figure S1.** Climate chamber temperatures during the three simulated mid-winter scenarios. Cold acclimating temperatures and spring growth temperatures were the same for all plants (upper panel) while mid-winter treatments (lower panel) included (1) high temperature fluctuation (warmfrost, long-dashed line), (2) low above freezing temperatures (mild, solid line), and (3) prolonged mild frost (frost, short-dashed line). Unfilled arrows indicate height measurements; the black arrow indicates the date of biomass harvest.


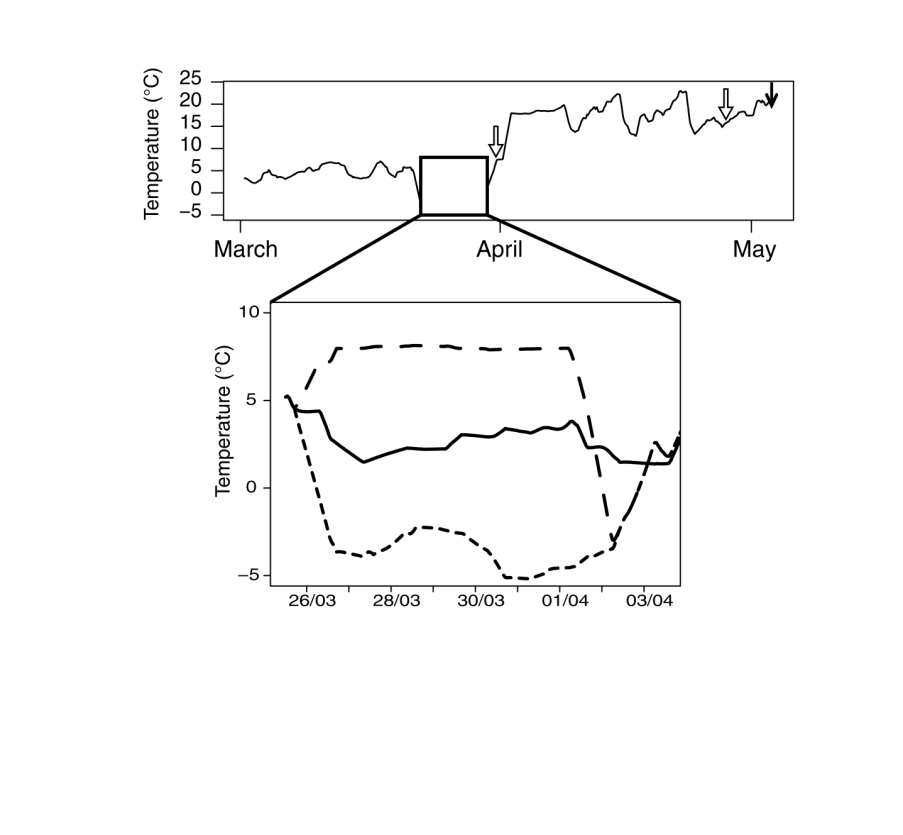


**Figure S2.** Climatic distances (based on bioclimatic data extracted from the Worldclim database) increase with increasing spatial distance between sampled populations of *Arrhenatherum elatius*.

**Figure S3.** Latitudinal patterns of population mean traits across treatments. Error bars indicate the standard error of the estimate. Note that for biomass the response differed depending on the winter scenario applied. For visualization of significant relationships only, linear regression lines (dashed) have been plotted. For probability of flowering, estimates from GLMMs were back transformed to actually represent probabilities.


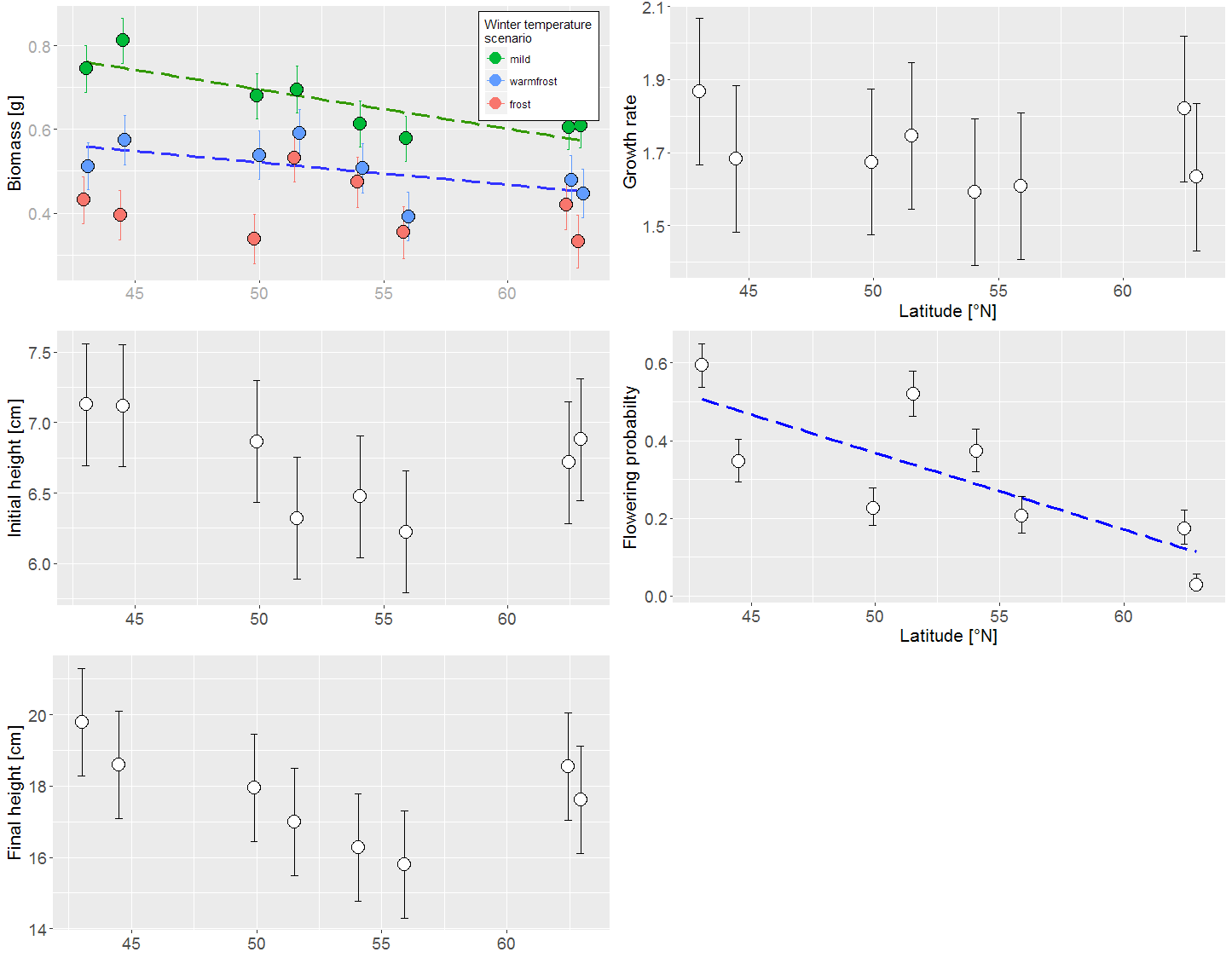


**Figure S4**. Difference between the posterior distribution of *Q*_ST_ and the simulated distribution of *Q*_ST_^n^ for trait means indicating potential adaptive differentiation, separated by winter temperature conditions experimentally applied. The dashed, red line represents the zero difference between *Q*_ST_ and *Q*_ST_^n^, i.e. the expectation under a gene flow-drift-only scenario. The dots indicate the mean and the error bars the 95 % credible interval of that difference.


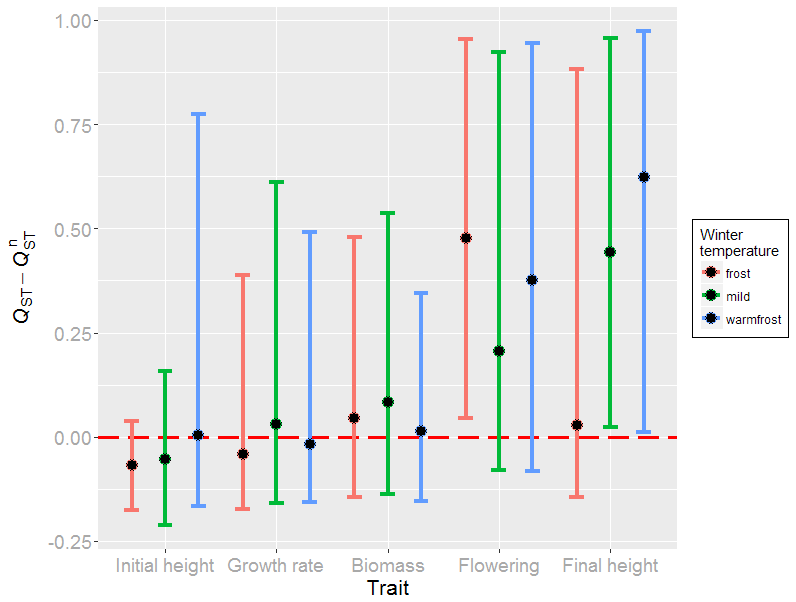


**Genetic and cross-environment correlations**

To assess whether the genetic basis for trait expression differed between the different mid-winter treatments, we estimated cross-environment genetic correlations as simple Pearson’s correlations between block corrected family means from each treatment. This method may result in downwardly biased estimates but provides a conservative test of whether a genetic correlation is different from zero (Astles et al. 2006).

Table 3. Across-treatment correlation coefficients for pairwise-comparisons of family trait means between different mid-winter treatments. A significant deviation from zero (*P* < 0.05) is indicated by bold numbers.

|  | Treatment comparison | | |
| --- | --- | --- | --- |
| *Trait* | *Mild vs. frost* | *Mild vs. warmfrost* | *Frost vs. warmfrost* |
| Biomass | 0.119 | **0.630** | **0.422** |
| Initial height | **0.672** | **0.644** | **0.488** |
| Final height | 0.212 | **0.666** | **0.328** |
| Relative growth rate | 0.089 | **0.400** | -0.015 |
| Probability of flowering | **0.375** | 0.229 | **0.601** |

**
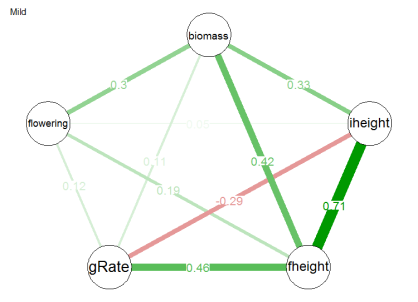

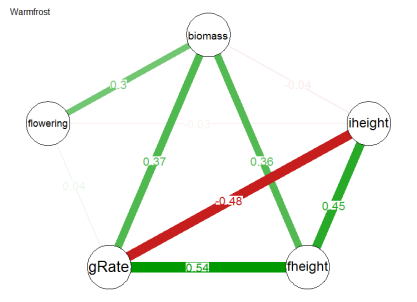

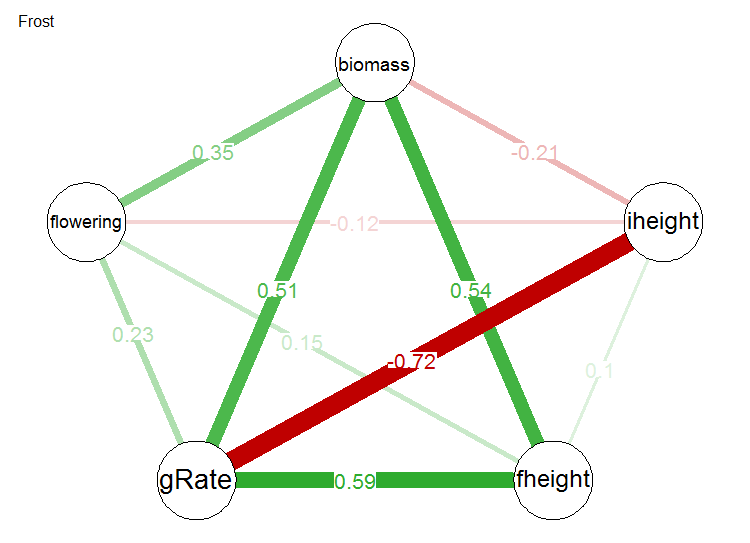
Figure S5**. Genetic correlations among family mean traits within different mid-winter treatments for biomass production, initial and final plant height (iheight and fheight, respectively), relative growth rate (gRate) and probability of flower production for the three mid-winter scenarios experimentally applied.

**Figure S6** . Windswept meadow dominated by the study species *Arrhenatherum elatius*.

**
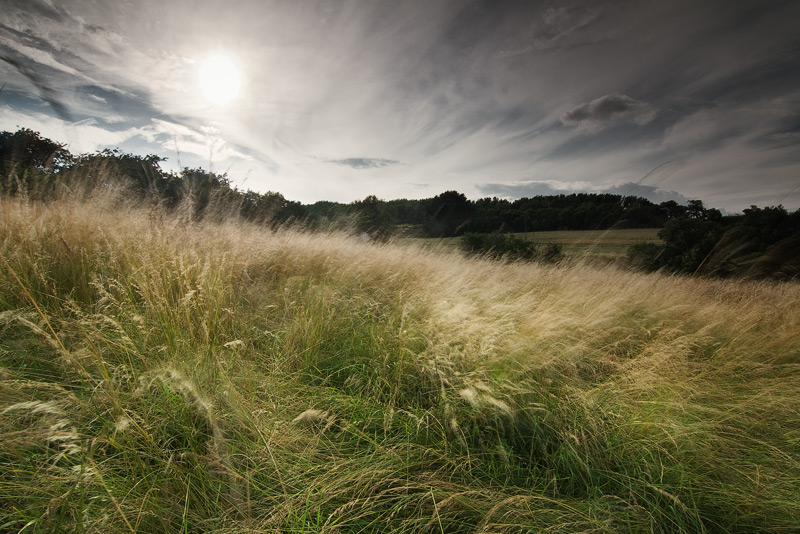
**

**References**

Astles, P. A., A. J. Moore, and R. F. Preziosi. 2006. A comparison of methods to estimate cross-environment genetic correlations. Journal of Evolutionary Biology **19**:114-122.

Gelman, A. 2006. Prior distributions for variance parameters in hierarchical models (comment on article by Browne and Draper). Bayesian Analysis **1**:515-534.

Hadfield, J. D. 2010. MCMC methods for multi-mesponse meneralized minear mixed models: The MCMC R package. Journal of Statistical Software **33**:1-22.

Nakagawa, S. and H. Schielzeth. 2010. Repeatability for Gaussian and non-Gaussian data: a practical guide for biologists. Biological Reviews of the Cambridge Philosophical Society **85**:935-56.
